# Interleukin-10 production at the early stage of infection with foot-and-mouth disease virus related to the likelihood of persistent infection in cattle

**DOI:** 10.1186/s13567-015-0276-y

**Published:** 2015-11-19

**Authors:** Zhidong Zhang, Claudia Doel, John B. Bashiruddin

**Affiliations:** State Key Laboratory of Veterinary Etiological Biology, Lanzhou Veterinary Research Institute, Xujiaping 1, Lanzhou, 730046 Gansu China; The Pirbright Institute, Ash Road, Pirbright, Woking, Surrey, GU24 0NF UK; DCD Consulting Ltd, Alton, Hants, GU34 5BG UK; JBBiologik, Farnham, Surrey, GU10 1DH UK

## Abstract

The factors leading to persistent infection of foot-and-mouth disease (FMD) virus in ruminants are not well defined. This paper provides evidence of the presence of interleukin-10 (IL-10) early in the course of infection (1–4 days) as a factor in the development of persistence of FMD virus in cattle. Results showed that serum IL-10 in carrier cattle infected with FMD virus type O (*n* = 4) was detected and peaked at 1 or 2 days post infection and rapidly declined thereafter. In contract, serum IL-10 levels in non-carrier cattle (*n* = 21) were very low or undetectable during the same period.

## Introduction, methods and results

Foot-and-mouth disease (FMD) virus is a small, non-enveloped single stranded, positive sense RNA virus (Picornaviridae family) and has seven serotypes: O, A, C, Asia 1, and Southern African Territories (SAT) 1, 2 and 3, all of which cause a highly contagious vesicular disease in cloven-hoofed animals [1]. After the acute phase of infection, which may be associated with clinical disease or with asymptomatic infection, up to 50% of FMD virus-infected cattle and other bovids may carry the virus in their pharyngeal regions for months or years without showing any clinical signs of disease, and may be critical to the epidemiology of FMD, at least at the wildlife domestic cattle interface (reviewed in [1, 2]). Carrier animals are defined as persistently infected animals from which FMD virus can be recovered from esophageal-pharyngeal fluid (Probang) for more than 28 days after infection [3]. FMD virus is eventually eliminated from carriers. However, the host factors that enable persistence or elimination are not known. Previous studies showed that the persistence with FMD virus was less likely to be established if the immune response to vaccination had developed sufficiently [4] and the rate of clearance of FMD virus from the pharyngeal region may be related to the likelihood of persistence [5, 6]. Recent studies have shown that FMD virus-induced immunosuppression during the acute stage of infection is not due to infection of the lymphocytes [7] but lack of T cell responsiveness via interleukin-10 (IL-10) signalling [8]. IL-10 is an immunoregulatory cytokine that can modulate immune processes associated with the anti-inflammatory response and the inhibition of cellular responses via a variety of mechanisms. It has been demonstrated that elevated levels of IL-10 production are associated with persistent infection with hepatitis C virus (HCV) [9, 10], HIV [11–13], Epstein–Barr virus [14] and lymphocytic choriomeningitis virus (LCMV) [15, 16]. Interferons (IFN) are known to have antiviral activity and are considered to be important in the initial host cell defence against virus infection. It has been demonstrated that replication of FMD virus is highly sensitive to type I and II IFNs and that porcine or bovine IFN added to supernatants of cell cultures inhibits FMD virus replication in vitro [17, 18] and clears FMD virus from persistently-infected cells [18]. The aim of the current study was to define the early IL-10 and IFN responses of cattle infected with the type O FMD virus and its relationship to the persistence of FMD virus.

To address this, serum samples collected at 0, 1, 2, 3, and 4 days post-infection (dpi) from 15 cattle of varying ages infected with the type O UKG/34/2001 virus and similarly from ten cattle infected with the type O SKR/1/2000 virus were used for detection of IL-10 and IFN protein. Detailed results regarding the clinical profiles are presented elsewhere [5, 6]. Briefly, all inoculated animals showed clinical signs of FMD with the development of fever and vesicles on all feet, and tongue. Viraemia peaked at 2–3 dpi, which was cleared by day 4 in all animals. All cattle had specific virus in their Probang samples for at least 7 days after virus exposure which was cleared from the pharynx at different rates. Four out of 25 cattle became carrier animals as evidenced by virus in their Probang samples after 28 dpi and later. The remaining 21 cattle did not develop the carrier state (Table 1). Statistical analyses were carried out by using a non-parametric test (independent samples t test using Excel). *P* < 0.05 was considered statistically significant.

**Table 1:** Cattle infected with foot-and-mouth disease virus were used in this study.

Animal ID	Age (days)	Route of infection	Carrier Status	Virus
VD34	273	Subepidermo-lingual	Non-carrier	FMDV O UKG34/2001
VD35	335	Subepidermo-lingual	Non-carrier	FMDV O UKG34/2001
VD40	243	Subepidermo-lingual	Carrier	FMDV O UKG34/2001
VD41	304	Subepidermo-lingual	Carrier	FMDV O UKG34/2001
VD44	91	Subepidermo-lingual	Non-carrier	FMDV O UKG34/2001
VD45	91	Subepidermo-lingual	Non-carrier	FMDV O UKG34/2001
VD48	91	Subepidermo-lingual	Non-carrier	FMDV O UKG34/2001
VD49	91	Subepidermo-lingual	Non-carrier	FMDV O UKG34/2001
VD36	335	Direct contact	Non-carrier	FMDV O UKG34/2001
VD37	243	Direct contact	Non-carrier	FMDV O UKG34/2001
VD42	243	Direct contact	Non-carrier	FMDV O UKG34/2001
VD43	213	Direct contact	Non-carrier	FMDV O UKG34/2001
VD46	91	Direct contact	Non-carrier	FMDV O UKG34/2001
VD47	91	Direct contact	Non-carrier	FMDV O UKG34/2001
VD50	91	Direct contact	Non-carrier	FMDV O UKG34/2001
VI56	274	Subepidermo-lingual	Carrier	FMDV O SKR 2000
VI57	152	Subepidermo-lingual	Non-carrier	FMDV O SKR 2000
VI58	274	Subepidermo-lingual	Carrier	FMDV O SKR 2000
VI59	274	Direct contact	Non-carrier	FMDV O SKR 2000
VI60	152	Direct contact	Non-carrier	FMDV O SKR 2000
VI61	152	Subepidermo-lingual	Non-carrier	FMDV O SKR 2000
VI62	152	Subepidermo-lingual	Non-carrier	FMDV O SKR 2000
VI63	152	Direct contact	Non-carrier	FMDV O SKR 2000
VI64	274	Direct contact	Non-carrier	FMDV O SKR 2000
VI65	274	Direct contact	Non-carrier	FMDV O SKR 2000

To analyze differences in humoral responses between carriers (*n* = 4) and non-carriers (*n* = 21), IgG antibodies to the structural proteins of FMD virus were quantified in sera using liquid phase blocking ELISA (LPBE) as described previously [19]. Antibody titre of < 1:45 is considered as negative. As shown in (Table 2), the cohort (*n* = 15) infected with the type O UKG/34/2001 had seroconverted to be FMD positive. Type-specific antibodies were detected in all inoculated animals by 4 dpi (Table 2) and in the majority of contact animals (VD36, VD37, VD42, VD43, VD46, VD47, VD50) by 6 dpi (Table 2). There are no significant difference in levels of serum IgG antibodies to the structural proteins of FMD virus between carrier cattle and non-carrier animals (*p* = 0.44, *p* > 0.05). Similar results were also observed in sera collected from the cohort infected with the type O SKR isolate (data not shown). The results obtained suggest that the IgG antibody response to structural proteins of FMD virus is not associated with the development of persistence in cattle, which is consistent with previous reports [1].

**Table 2:** Antibody response in cattle infected with foot-and-mouth disease virus.

Animal ID	Days post inoculation											
	0	1	2	3	4	5	6	7	9	15	21	28
VD34	Neg	Neg	Neg	Neg	128	1448	≥2896	>2048	≥2896	≥2896	>2048	>2048
VD35	Neg	Neg	Neg	Neg	362	≥2896	≥2896	>2048				
VD40	Neg	Neg	Neg	Neg	128	1024	1448	>2048	1448	1448	1448	1448
VD41	Neg	Neg	Neg	Neg	90	1448	1448	>2048	1448	2048	>2048	>2048
VD44	Neg	Neg	Neg	45	1024	≥2896	≥2896	>2048	1448	≥ 2896	>2048	>2048
VD45	Neg	Neg	Neg	Neg	128	1448	1448	>2048	1448	2048	>2048	>2048
VD48	Neg	Neg	Neg	Neg	128	≥2896	≥2896	>2048	ND	ND	>2048	>2048
VD49	Neg	Neg	Neg	Neg	45	Neg	1024	>2048				
VD36	Neg	Neg	Neg	Neg	Neg	Neg	362	1448				
VD37	Neg	Neg	Neg	Neg	Neg	Neg	512	1448	2048	≥2896	>2048	>2048
VD42	Neg	Neg	Neg	Neg	Neg	Neg	724	>2048	≥2896	≥2896	>2048	>2048
VD43	Neg	Neg	Neg	Neg	Neg	Neg	256	2048	1448	≥2896	2048	1148
VD46	Neg	Neg	Neg	Neg	Neg	1448	Neg	Neg	90	≥2896	>2048	
VD47	Neg	Neg	Neg	Neg	Neg	Neg	Neg	Neg	Neg	1024	>2048	>2048
VD50	Neg	Neg	Neg	Neg	Neg	Neg	Neg	362	2048	≥2896		

**Figure 1:**
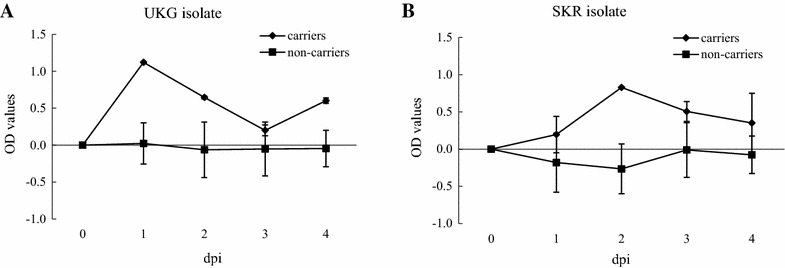
**Interleukin-10 (IL-10) in sera of cattle during the acute phase of FMD virus infection.** 25 cattle were assayed for circulating IL-10 by ELISA from day 0 to day 4 post infection. **A** Two carrier cattle and 13 non-carrier cattle infected with FMD virus O UKG34/2001; **B** Two carrier cattle and eight non-carrier cattle infected with FMD virus O SKR/2000; Error bars show STDEV of the mean.

To investigate if elevation of serum IL-10 during the acute infection correlates with development of persistent infection in cattle, IL-10 was measured basically following the method of Kwong et al. [20]. Briefly, enzyme-linked immunosorbent assay (ELISA) plates were coated with anti-IL-10 and each cattle serum was tested in duplicate. The assay was repeated three times. As shown in Figure 1, the elevation of serum IL-10 during the acute phase of FMD virus infection with either type O UKG or type O SKR isolates (from 1 to 4 dpi) was only observed in sera collected from carrier cattle (*n* = 4). In the carrier group, serum IL-10 was detected and peaked at 1 or 2 dpi and rapidly declined afterwards. In contrast, it was evident that levels of IL-10 in sera from the non-carrier group (*n* = 21) were much lower or undetectable. Comparison of serum IL-10 levels between carrier and non-carrier cattle showed that levels from carrier cattle during early infection were significantly higher than in non-carrier animals (*p* = 0.025, *p* < 0.05), suggesting an association of IL-10 response during the acute phase of infection with a common outcome of FMD disease (i.e. persistence) in cattle. Age at infection is known to influence the establishment of viral persistence [21, 22] and bacterial infection [23]. It has also been shown that infected calves (4–9 weeks of age) with FMD virus did not always developed clearly visible clinical signs of FMD [24] as observed in infected adult cattle. Interestingly, persistent infection was previously reported to only occur in a group of cattle which is older than 8 months at infection [25]. Similar observation was found in this study. No animal at age of younger than 6 months at infection (*n* = 12) became carrier (Table 1), which is correlated with the observations that no elevation of serum IL-10 during the acute phase of FMD virus infection with either type O UKG or type O SKR isolates (from 1 to 4 dpi), IL-10 ranging from −0.07 ± 0.21 to −0.08 ± 0.12 was observed in sera collected from these young animals, further suggesting IL-10 as a contributing factor in the likelihood of persistence of FMD virus. In contrast, when the IFN-gamma response was tested using the same serum samples, the results did not correlate with the outcome of disease (i.e. carrier or non-carrier) although increases in levels of serum IFN-gamma occurred in some animals after infection (data not shown).

## Discussion

The results obtained in this study suggest that the humoral IgG response to structural proteins of FMD virus is not associated with the development of persistence in cattle, however, the previous study showed that persistence with FMD virus was less likely to be established if effective immune response to vaccination had developed [4]. IL-10 is an immunoregulatory cytokine which is widely acknowledged to contribute to the inhibition of immune responses via a variety of mechanisms. Many studies with other viruses have indicated that IL-10 initiates T cell inactivation during viral infection and have concluded that this can lead to viral persistence [1, 9–16]. The present paper provides evidence of the association of IL-10 response during the acute phase of infection with a common outcome of FMD disease (i.e. persistence) in cattle. Although much is still unclear how IL-10 regulates the host immune response, it is possible that the relatively high IL-10 response causes a delay in or the inhibition of both the type-1 response and T cell activation until it is “too late” to arrest the disease process, resulting in incomplete clearance of clearing the virus, leading to the persistence. Of course, extrapolation of the results from this study on cattle is always questionable because persistence with FMD virus also occurs in small ruminants [1]. Furthermore, the elevation of serum IL-10 levels raises the question of why IL-10 induction did not occur in all infected cattle and whether IL-10 is the only member of this cytokine family that could influence the outcome of infection with FMD virus. The apparent relationship between the development of the carrier state and IL-10 response prompts further investigation.

## Abbreviations

FMD: foot-and-mouth disease
IL-10: interleukin-10
ELISA: enzyme-linked immunosorbent assay
dpi: days post-infection
IFN: interferon
